# Das Potenzial von Instant Messaging für soziale Beziehungen von Menschen 65+

**DOI:** 10.1007/s00391-021-01911-w

**Published:** 2021-06-25

**Authors:** Cora Pauli, Rhea Braunwalder, Veronika Hämmerle, Julia Reiner, Sabina Misoch

**Affiliations:** Institut für Altersforschung, OST – Ostschweizer Fachhochschule, Rosenbergstraße 59, 9001 St. Gallen, Schweiz

**Keywords:** Alter, Sozioemotionale Selektivitätstheorie, Soziales Kapital, WhatsApp, Older adults, Socioemotional selectivity theory, Social capital, WhatsApp

## Abstract

**Hintergrund und Fragestellung:**

Aktuelle Daten aus der Schweiz zeigen, dass zunehmend auch ältere Personen (65+) Instant-Messaging-Dienste nutzen. Am Beispiel von WhatsApp soll untersucht werden, ob und wie sich die Nutzung von WhatsApp auf unterschiedliche Formen des sozialen Kapitals älterer Menschen auswirkt, und ob die sozioemotionale Selektivitätstheorie auch im digitalen Raum angewendet werden kann.

**Untersuchungsmethoden:**

Es wurde eine qualitative Studie mit 30 WhatsApp-Nutzer(innen) der Altersgruppe 65+ durchgeführt. Die Befragung fußte auf der Erstellung von egozentrierten Netzwerkkarten und der Durchführung von Interviews anhand eines semistrukturierten Leitfadens.

**Ergebnisse:**

WhatsApp wird v. a. für die Kommunikation mit nahestehenden Personen genutzt. Die Nutzung von WhatsApp vereinfacht die Beziehungspflege, erhöht die Kontakthäufigkeit und kann zur Intensivierung von Beziehungen führen. Die Nutzung von WhatsApp kann zudem das Zugehörigkeitsgefühl zu Gruppen stärken. Das Medium wird als sehr niederschwellig beschrieben und erlaubt Spontaneität.

**Diskussion:**

Die sozioemotionale Selektivitätstheorie ist auch im digitalen Raum anwendbar. Via WhatsApp wurden tendenziell positive Inhalte mit emotional bedeutsamen Personen des Netzwerks geteilt. Die Nutzung von WhatsApp erhöht sowohl das „bonding social capital“, da Beziehungen vertieft und verstärkt werden, als auch das „maintaining social capital“, da geografische Distanzen überbrückt werden können.

## Abstract (en)

**Background and research question:**

Current data from Switzerland show that an increasing number of older adults (65+ years) use instant messaging services. We examined whether and how the use of WhatsApp affects different forms of older people’s social capital and whether the socioemotional selectivity theory can be applied in digital environments.

**Research methods:**

A qualitative study was conducted with 30 WhatsApp users in the age group 65+. The survey was based on data from ego-centered network maps and interviews using a semistructured interview guide.

**Results:**

WhatsApp is mainly used for communication with close persons. The use of WhatsApp simplifies relationship management, increases the frequency of contact and can thus lead to intensified relationships. The use of WhatsApp can strengthen the sense of group belonging. The media is described as very low-threshold and enables spontaneity.

**Discussion:**

The socioemotional selectivity theory can also be applied in digital environments. Via WhatsApp, positive contents are shared with emotionally significant people in the social network. The use of WhatsApp increases both bonding social capital as relationships are deepened and strengthened and maintaining social capital as geographical distances can be bridged.

In der Schweiz ist WhatsApp ist der am häufigsten genutzte Instant-Messaging-Dienst, und der Anteil älterer Menschen, die Instant Messaging nutzen, steigt stetig an. Obwohl es in der Forschung Hinweise gibt, dass sich die Digitalisierung positiv auf soziale Beziehungen älterer Menschen auswirken kann, gibt es noch wenig empirische Evidenz dazu. Um diese Forschungslücke zu schließen, wurde in der deutschsprachigen Schweiz eine qualitative Studie mit WhatsApp-Nutzer(innen) der Altersgruppe 65+ durchgeführt.

## Hintergrund und Fragestellung

Der demografische Wandel und die Digitalisierung sind grundlegende Trends in modernen Industriegesellschaften. Aktuelle Studien zeigen, dass europaweit ein digitaler Graben hinsichtlich des Alters von Internetnutzer(innen) besteht (z. B. [9, 14, 26, 29]). Gleichzeitig zeichnet sich in der Schweiz bei älteren Menschen eine deutliche Zunahme der Nutzung des Internets ab [19, 25], auch über mobile Geräte [4]. Vor diesem Hintergrund ist eine Betrachtung der sozialen Interaktion via Smartphone von älteren Menschen am Beispiel der Nutzung von Instant Messengers (im Folgenden IM genannt) interessant, zumal bislang wenig Studien zu dieser Altersgruppe existieren [23]. Instant-Messaging-Dienste erlauben es den Nutzenden, via Smartphone online in Echtzeit Text‑, Bild‑, Video- und Tondateien auszutauschen.

Empirische Studien belegen, dass sich soziale Netzwerke im Laufe des Lebens verkleinern (z. B. [5, 6, 16]). Gemäß der Theorie der sozioemotionalen Selektivität (z. B. [5, 15]) beruht dieses Phänomen auf einem strategischen Selektions- bzw. Auswahlprozess: Während jüngere Menschen eher zur Erweiterung ihres Netzwerks durch neue Sozialkontakte streben, verkleinern sich soziale Netzwerke mit zunehmenden Alter zugunsten der Qualität der Sozialkontakte. Vor dem Hintergrund einer sich verkürzenden Zukunftsperspektive fokussieren sich ältere Menschen stärker auf emotional bedeutsame und befriedigende Kontakte. Emotionale Intimität und das Gefühl von Zugehörigkeit sind Voraussetzungen für emotional befriedigende Beziehungen und entstehen in Kontakten zu sehr nahestehenden Personen [5].

Das soziale Kapital ist ein Konzept, um soziale Beziehungen zu betrachten. Es kann definiert werden als „Gesamtheit der aktuellen oder potenziellen Ressourcen, die mit dem Besitz eines dauerhaften Netzes von mehr oder weniger institutionalisierten *Beziehungen* gegenseitigen Kennens oder Anerkennens verbunden sind“ [3]. Soziales Kapital wird durch individuelle soziale Beziehungen erzeugt [10] und beinhaltet Dimensionen wie soziale Verbundenheit, soziale Unterstützung und Teilhabe [2]. Es ist als Ressource im Alter besonders wichtig, da es positive Effekte auf Wohlbefinden und Gesundheit haben kann [2]. Soziales Kapital ist ein mehrdimensionales Konzept [11] und kann in die Formen „bridging social capital“ und „bonding social capital“ ausdifferenziert werden [22]. „Bridging social capital“ basiert auf sozialen Beziehungen zwischen weiter entfernen Netzwerkpartner(innen) und ermöglicht den Austausch praktischer Informationen [8, 11]. „Bonding social capital“ entsteht in engen Beziehungen und kann in emotionale oder soziale Unterstützung münden [1, 28]. Ellison et al. [8] ergänzen die beschriebenen Kapitalsorten mit „maintaining social capital“, welches die Möglichkeit beschreibt, mittels sozialer Medien trotz geografischer Distanz Beziehungen zu pflegen.

Im vorliegenden Artikel wird gestützt auf die sozioemotionale Selektivitätstheorie der Frage nachgegangen, ob sich die Nutzung von WhatsApp aus subjektiver Sicht der Nutzenden auf ihre sozialen Beziehungen auswirkt. Weiter wird untersucht, ob die sozioemotionale Selektivitätstheorie auch im digitalen Raum – für die Nutzung von WhatsApp – anwendbar ist. Zudem wird untersucht, auf welche der genannten sozialen Kapitalformen sich die Nutzung von WhatsApp auswirkt. Mit Blick auf die sozioemotionale Selektivitätstheorie kann von von der besonderen Wichtigkeit des „bonding social capital“ für alte Menschen ausgegangen werden, da dieses den Nährboden für emotional bedeutsame und befriedigende Beziehungen bildet.

Die im Folgenden beschriebenen Daten basieren auf einem Forschungsprojekt, welches finanziert vom Schweizerischen Nationalfonds zwischen 2018 und 2020 in der Schweiz durchgeführt wurde. WhatsApp wurde ausgewählt, da dieser IM-Dienst in der Schweiz zum Zeitpunkt der Untersuchung der am häufigsten genutzte war [29].

## Studiendesign und Untersuchungsmethoden

**Figure 1:**
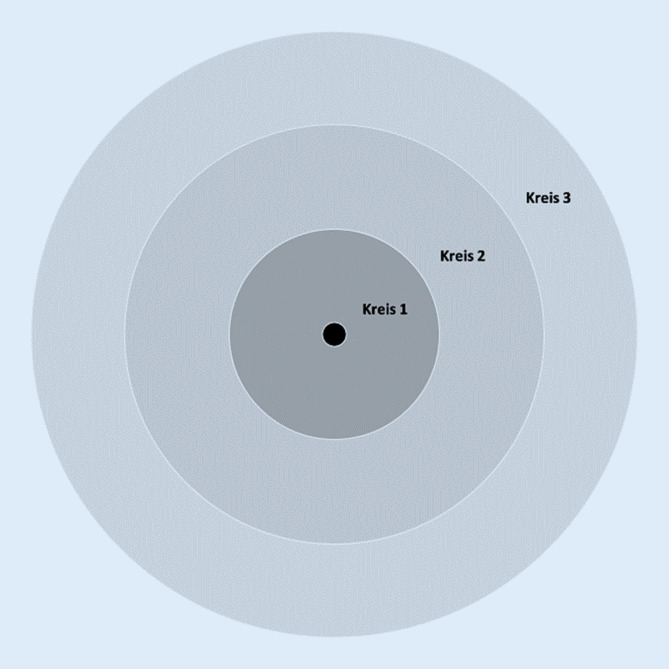

Das Forschungsvorhaben war als explorative, qualitative Studie angelegt und wurde mit WhatsApp-Nutzenden im Alter von 65+ durchgeführt. Diese Altersgrenze markiert in der Schweiz das Erreichen des Pensionsalters und wird auch in anderen Studien für die Bezeichnung der Lebensphase „Alter“ verwendet (z. B. [25]). Es wurden explizit aktive und regelmäßige WhatsApp-Nutzer(innen) rekrutiert, da die Frage nach möglichen Auswirkungen der Nutzung auf soziale Beziehungen im Fokus der Studie stand. Die qualitative „Face-to-face“-Befragung erfolgte zwischen März und Juli 2019 und fußte auf der Erstellung von egozentrierten Netzwerkkarten [12] und der Durchführung von leitfadengestützten Interviews [18]. Zur Erhebung des sozialen Netzwerks wurde in Anlehnung an Kahn und Antonucci [12] eine Karte mit drei konzentrischen Kreisen vorbereitet (Abb. 1). Die Teilnehmenden wurden gebeten, aus dem Gedächtnis Personen zu nennen, mit denen sie sich emotional verbunden fühlten und deren Namen (anonymisiert) auf Klebezettel zu notieren^1^. In einem nächsten Schritt positionierten die Befragten die Klebezettel je nach Grad der emotionalen Verbundenheit^2^ auf der Karte (Kreis 1: sehr starkes Verbundenheitsgefühl bis Kreis 3: schwaches Verbundenheitsgefühl) und markierten Personen, mit denen WhatsApp genutzt wurde.

Im Anschluss wurde ein leitfadengestütztes Interview durchgeführt. Dies beinhaltete Fragen zu den mit Netzwerkpartner(innen) via WhatsApp ausgetauschten und nichtausgetauschten Inhalten, zu den genutzten WhatsApp-Funktionen, zu den Auswirkungen auf soziale Beziehungen (Beziehungsqualität, Zugehörigkeitsgefühl zu Einzelnen und Gruppen), zu den Nutzungsgewohnheiten, Vorteilen und Nachteilen (z. B. im Vergleich mit Kommunikationskanälen wie E‑Mail oder Telefonanrufen) und zur Nutzungsgeschichte von WhatsApp.

Die qualitativen Interviews wurden auditiv aufgezeichnet und verbatim transkribiert^3^. Das Interviewmaterial wurde inhaltsanalytisch ausgewertet [17]. Die Dateneingabe der Netzwerkkarten erfolgte über SPSS. Die Daten wurden deskriptiv ausgewertet. Der Datensatz wurde außerdem auf Geschlechtsunterschiede hinsichtlich der Netzwerkgrößen überprüft.

Rekrutiert wurde über das Kontaktnetz des Instituts für Altersforschung der Ostschweizer Fachhochschule und mittels Schneeball-Sampling. Eine Übersicht über das Sample bietet Tab. 1. Eher überproportional vertreten waren Personen mit einem hohen sozioökonomischen Status.

**Table 1:** 

Merkmal		*n*
Geschlecht	Frauen	15
	Männer	15
Alter (gruppiert)	65–70	13
	70–80	14
	Über 80	3
Höchster abgeschlossener Bildungsgrad	Tertiärstufe	20
	Sekundärstufe	10
Wohnform	Alleine	9
	Mit Partner(in)	20
	Sonstiges	1
Kinder	Ja	22
	Nein	8
Monatliches Haushaltseinkommen in CHF	10.000+	7
	7500–10.000	3
	5500–7500	8
	3500–5500	5
	1500–3500	3
	Keine Angabe	4

## Ergebnisse

### Soziale Netzwerke

Die Befragten platzierten im Durchschnitt 8 Personen im Netzwerkkreis 1, neun im Netzwerkkreis 2 und 7 im Netzwerkkreis 3 (Tab. 2). Durchschnittlich nutzten die Befragten innerhalb ihrer Netzwerke mit 18 (SD = ±7,2) Personen WhatsApp und mit 5 (SD = ±3,4) Personen kein WhatsApp.

**Table 2:** 

	*n*	M	SD	Min	Max
Netzwerkkreis 1	30	7,9	4,8	2	21
Netzwerkkreis 2	30	8,7	4,6	3	22
Netzwerkkreis 3	30	6,8	4,0	0	15
Gesamtnetzwerk	30	23,4	7,9	11	45

Mittels Mann-Whitney-U-Test wurde untersucht, ob sich die Gesamtnetzwerkgröße sowie die Größen der einzelnen Netzwerkkreise von Frauen und Männern unterscheiden. Das Signifikanzniveau wurde mit *p* < 0,05 festgelegt. Es ergaben sich keine signifikanten Unterschiede zwischen Frauen und Männern hinsichtlich der Größe des Gesamtnetzwerkes (*p* = 0,806) sowie der Größe der Netzwerkkreise 1 (*p* = 0,148), 2 (*p* = 0,624) und 3 (*p* = 0,486).

Die Befragten gaben an, dass ihr soziales Netzwerk in den letzten Jahren weitestgehend konstant geblieben ist und die Nutzung von WhatsApp nicht zur Erweiterung ihres sozialen Netzwerks beitrug. Im Vordergrund stand die Pflege von bestehenden Kontakten bzw. das Reaktivieren von Kontakten.

### Auswirkungen der WhatsApp-Nutzung auf soziale Beziehungen

WhatsApp wurde von den Befragten regelmäßig (mehrmals täglich oder mehrmals wöchentlich) genutzt. Die Analyse der Interviews zeigte, dass sich als Folge der Nutzung von WhatsApp bestehende Beziehungen aus den Netzwerkkreisen 1 und 2 bezüglich Kontakthäufigkeit und empfundener Nähe intensivierten. Zwischen den Netzwerkkreisen 1 und 2 zeigten sich keine wesentlichen Unterschiede hinsichtlich der ausgetauschten Inhalte und der Auswirkungen der WhatsApp-Nutzung auf die sozialen Beziehungen. Auf die Qualität von Beziehungen aus dem Netzwerkkreis 3 hatte WhatsApp keine Auswirkungen. Folgende Faktoren führten zu der Intensivierung sozialer Beziehungen in den Netzwerkkreisen 1 und 2:

#### Vereinfachung der Beziehungspflege, Zunahme der Kontakthäufigkeit und der Beziehungsintensität.

In den Interviews wurde oft geschildert, dass die Nutzung von WhatsApp das Kontakthalten und die Beziehungspflege v. a. in den beiden inneren Kreisen des Netzwerks vereinfachte. WhatsApp eigne sich für das spontane Teilen kleiner „Kommunikationsfetzen“ wie z. B. Grüßen, Ferienfotos und Alltagsimpressionen etc. Die Vereinfachung der Beziehungspflege wurde auch im Zusammenhang mit geografisch entfernten Personen betont: „Mein Sohn und seine Familie waren im Herbst im Südtirol in den Ferien und waren wandern. Und dann haben sie manchmal ein Bild vom Wandern oder vom Picknicken oder so geschickt, einfach so. Und daran hatte ich sehr große Freude. Dann sehe auch ein wenig, wie sie sich entwickeln [Enkelkinder], weil ich sie sonst nicht so viel sehe. Wie sie groß werden“ (Frau E., 79-jährig). Bei im Ausland lebenden Freunden(innen) und Verwandten ermöglichte WhatsApp eine kontinuierlichere Kommunikation im Vergleich zur Telefonie, da Kosten oder der Zeitunterschied keine Rolle spielten.

Eine Botschaft per WhatsApp zu verschicken, war aus Sicht der Befragten niederschwelliger als anzurufen oder eine E‑Mail zu versenden: „Und ich habe das Gefühl, ich störe den anderen weniger, wenn ich ihm ein WhatsApp schicke, als wenn ich jetzt telefoniere. Es braucht ja Präsenz, das Telefonieren. Und beim WhatsApp kann man das noch etwas hinausziehen. Mich dünkt es eigentlich eine diskrete Art von Anteilnehmen am gegenseitigen Geschehen“ (Frau E., 69-jährig). Dies führt zu häufigeren Kontaktnahmen und zu mehr Nähe der Netzwerkpartner(innen): „Ja, ich denke, das ist eigentlich sowieso so. Je mehr Kontakt man miteinander hat, desto näher steht man einander“ (Herr B., 69-jährig). Das häufigere Teilen von Inhalten und die kontinuierliche Erreichbarkeit der Netzwerkpartner(innen) führten zu einer Zunahme der gefühlten Intensität von Beziehungen: „Man fühlt sich irgendwie näher bei dieser Person. Also sie ist ja jederzeit erreichbar, oder. Es ist, also es ist ein Gewinn, ein Plus an Nähe. Und weil es so einfach ist, gibt es häufigere Kontaktaufnahmen, und das wirkt sich auch positiv aus“ (Herr R., 76-jährig). WhatsApp wurde auch genutzt, um Freunde/Freundinnen in schwierigen Situationen zu unterstützen (z. B. nach einer Operation). Auch hier wurde die Unaufdringlichkeit als Vorteil geschildert: Der/die Empfänger(in) schaut die Nachricht an und reagiert, wenn der Zustand es erlaubt.

#### Erweiterte Möglichkeiten zur sozialen Teilnahme.

Es wurde eine große Bandbreite von Inhalten ausgetauscht: Alltagsereignisse, Lebensereignisse (z. B. erste Schritte des Enkelkindes). Diese Inhalte wurden vor der Nutzung von WhatsApp nicht in derselben Häufigkeit oder gar nicht ausgetauscht und ermöglichten mehr Anteilnahme am Leben anderer, was zu größerer Nähe führte. Die visuellen Kommunikationsmöglichkeiten spielten eine wichtige Rolle: „Ich denke schon, dass es [Fototausch via WhatsApp] einem hilft, ein bisschen mehr in andere hineinzusehen. Wie andere sich fühlen, wenn man an einer anderen Erlebniswelt teilnimmt. Ich denke, das gibt einem schon noch mehr Einblick. Und durch das kann es vielleicht auch den Kontakt ein bisschen festigen, intensivieren, stärker machen“ (Herr B., 69-jährig). Die Befragten nutzten Textnachrichten und Fotos, es wurden aber auch Emoticons, Sprachnachrichten und Videos verschickt.

#### Stärkung der familiären Gruppenzugehörigkeit.

Innerhalb von Familien benutzten viele der Befragten unterschiedliche WhatsApp-Gruppen: eine für die Kernfamilie, eine für die Kernfamilie plus Schwiegerkinder, eine nur für Geschwister etc. In den Familienchats wurden Beratung über Geschenke, Planung von Treffen, Erinnerungen, besondere Momente aus dem Familienalltag etc. besprochen. Die Befragten beschrieben, dass Familienchats sich positiv auf die familiären Bande und das Gefühl der Zugehörigkeit auswirkten. Weiter wurde die Möglichkeit, via Gruppenchat Termine einfach zu koordinieren, als praktisch empfunden.

Insgesamt wurde der Austausch via WhatsApp in den beiden inneren Kreisen des Netzwerks als bereichernd und erfreulich beschrieben: „Einfach durch diese Häufigkeit, wo man das nutzt, das ist, dass ist immer, jedes Mal so, wie soll ich sagen, wie eine Streicheleinheit, wo ich entweder bekomme oder ich gebe. Ich denke an dich, ich schicke dir ein WhatsApp. Oder ich bekomme eins“ (Herr R., 76-jährig). Auch, weil v. a. Positives versendet wurde: „Ja. Ich nutze es gern, das WhatsApp. Weil ich finde es so ein …, so ein leichtes, positives Medium und auch ein fröhliches (…)“ (Frau A., 67-jährig).

### Nachteile und Grenzen des Mediums

Einige der Befragten äußerten, dass sie manchmal auch zu viele für sie unwichtige oder nichtinteressante Nachrichten und Bilder empfingen (z. B. innerhalb von Gruppenchats). In Ausnahmen wurde der Zeitdruck erwähnt, das Gefühl, schnell auf Nachrichten antworten zu müssen. Auch wurde die Befürchtung geäußert, durch die Nutzung von WhatsApp könnten andere Formen des Kontakts wie Telefonate zweitrangig werden. Als Tabu wurde das Verhandeln von Konflikten oder Kondolenzbezeugungen genannt. Betreffend Datenschutz wurden diffuse Bedenken geäußert, dass mit der Nutzung von IM der Datenschutz nicht gewährleistet sei. Um eigene Daten bzw. die Daten Dritter zu schützen wurde z. B. auf die Übermittlung persönlicher Daten wie Bankkontoverbindungen und Passwörtern sowie auf den Versand von Fotos unbekleideter Enkelkinder verzichtet.

## Diskussion

Mit der Nutzung von WhatsApp wird ein zusätzlicher Kommunikationsraum erschlossen, der in erster Linie mit emotional bedeutsamen Personen (Netzwerkkreise 1 und 2) genutzt wird. Eine Vielfalt von Inhalten kann auf einfache Weise geteilt werden, was diese Beziehungen bereichert, die Präsenz enger Netzwerkpartner(innen) erhöht und in der Folge die soziale Verbundenheit stärkt. Die in der sozioemotionalen Selektivitätstheorie beschriebenen Mechanismen sind auch in der digitalen Kommunikation sichtbar. Die starke Ausrichtung der WhatsApp-Kommunikation auf positive Inhalte weist Parallelen zu vergangenen empirischen Studien in Verbindung mit der sozioemotionalen Selektivitätstheorie auf, wonach ältere Personen sich stärker auf positive Inhalte und Aspekte konzentrieren [7] und wurde so auch in anderen Studien zur WhatsApp-Nutzung älterer Menschen beobachtet [24]. IM scheint ein geeignetes Instrument dafür zu sein, emotional befriedigende Beziehungen zu pflegen und zu vertiefen.

Das „bonding social capital“ kann als Nährboden für die aus Sicht der sozioemotionalen Selektivitätstheorie wesentlichen sozialen Bedürfnisse älterer Menschen betrachtet werden. Die WhatsApp-Kommunikation führt durch die gesteigerten Möglichkeiten der gegenseitigen Teilhabe zu einer Intensivierung und Verdichtung – sowohl im qualitativen als auch im quantitativen Sinn – von engen Beziehungen und damit zur Stärkung des „bonding social capital“. Dies dank der Spontaneität und der Niederschwelligkeit des Mediums und der Möglichkeit, Emotionalität und Intimität in der Kommunikation mit nahestehenden Personen herzustellen [13]. Voraussetzung dafür ist, dass sich die Kommunikationspartner(innen) im realen Leben nahestehen, um kurze, leichte und spontane „Kommunikationsfetzen“ wirkungsvoll einzusetzen [21]. Wie die Studienergebnisse zeigen, ist die Nutzung von WhatsApp für die intergenerationelle Kommunikation innerhalb der Familie wichtig und kann zur Stärkung der Familienbande beitragen [20, 27].

WhatsApp-Nachrichten können geografische Distanzen überbrücken und das Gefühl von Nähe schaffen und so der Aufrechterhaltung von sozialen Beziehungen im Sinne von „maintaining social capital“ dienen. Ebenfalls geben unsere Ergebnisse Hinweise darauf, dass im Fall von Veränderungen der Lebenssituation WhatsApp bei der Reaktivierung von sozialem Kapital unterstützen kann, wie z. B. der Wiederaufnahme des Kontaktes zu ehemaligen Schulfreunden(innen).

Die Nutzung von IM hat auch Kehrseiten, wie etwa den Empfang von irrelevanten Nachrichten und den Druck, schnell auf Nachrichten reagieren zu müssen [20]. Dies wurde von einigen Befragten als störend empfunden. Auch das Thema Datenschutz wurde wegen des Gefühls von Kontrollverlust über die eigenen Daten als Nachteil benannt. Die Problematik des Datenschutzes Dritter wurde nur von einer Befragten explizit adressiert; zu dieser Thematik bestand wenig Sensibilität. Da gerade der Austausch von Enkelbildern als übergenerationelles Kommunikationsverhalten und als wesentlicher und mit Freude verbundener Kommunikationsinhalt geschildert wurde, kann das damit verbundene Dilemma der Gefährdung von Privatsphäre schwer gelöst werden.

Die Resultate zeigen, dass die Nutzung von IM, trotz der geschilderten Nachteile, für ältere Erwachsene eine wichtige Ressource sein kann, weil die Verbundenheit mit dem enger werdenden Kreis von nahestehenden Personen gestärkt werden kann und mit Freude im Alltag verbunden ist.

## Limitationen

An der Studie haben vornehmlich Menschen mit einem hohen sozioökonomischen Status und einem stabilen sozialen Netzwerk teilgenommen. Der Einbezug von Personen mit niedrigeren sozioökonomischen, weniger konstanten Lebenssituationen oder sehr kleinen Netzwerken wäre für zukünftige Studien interessant und würde zu einer besseren theoretischen Sättigung der Ergebnisse beitragen.

Da ausschließlich aktive WhatsApp-Nutzer(innen) befragt wurden, können keine Aussagen zum Thema Nutzungs- und Zugangsbarrieren gemacht werden. Für ein umfassenderes Verständnis der Thematik Alter und Techniknutzung bzw. Zugang zur Digitalisierung und Chancengleichheit müssten auch Personen berücksichtigt werden, die nicht an der Digitalisierung teilhaben.

## Schlussfolgerung/Fazit für Praxis

- Instant-Messaging-Dienste machen es möglich, positive und emotional wichtige Erlebnisinhalte zu generieren und mit anderen zu teilen und können so den Alltag älterer Menschen bereichern.
- Instant-Messaging-Dienste können für ältere Menschen ein Werkzeug dafür sein, bedeutsame soziale Beziehungen zu pflegen und zu vertiefen und damit sozioemotionale Bedürfnisse zu befriedigen und das soziale Kapital zu stärken.
- Durch die Nutzung von Instant-Messaging-Diensten wird die Kontaktpflege zu geografisch weit entfernt lebenden sozialen Kontakten vereinfacht und die Reaktivierung alter Freundschaften begünstigt.
- Wenn ältere Bevölkerungsgruppen dieselben digitalen Kommunikationskanäle nutzen wie jüngere, kann der Kontakt zwischen Generationen vereinfacht und gefördert werden.
- Eine stärkere Sensibilisierung und Aufklärung zum Thema Datenschutz/Datenschutz Dritter wäre wünschenswert, und entsprechende Empfehlungen könnten z. B. von Altersorganisationen bereitgestellt werden.

## Footnotes

1 Einige Befragte nahmen dazu ihre WhatsApp-Kontaktlisten zu Hilfe.

2 Der Grad der emotionalen Verbundenheit wurde bei der Erhebung von den Forschenden folgendermaßen beschrieben: Zum Kreis 1 zählen Personen, ohne die man sich das Leben schwer vorstellen könnte und mit denen sehr persönliche Fragen besprochen werden; zum Kreis 2 zählen Personen, die ebenfalls wichtig sind, aber weniger essenziell. Zum Kreis 3 zählen Personen, mit denen ein loser/gelegentlicher Kontakt besteht.

3 Die anonymisierten Transkripte sind online unter DOI 10.5281/zenodo.3753978 einsehbar.
